# District decision-making for health in low-income settings: a feasibility study of a data-informed platform for health in India, Nigeria and Ethiopia

**DOI:** 10.1093/heapol/czw082

**Published:** 2016-09-01

**Authors:** Bilal Iqbal Avan, Della Berhanu, Nasir Umar, Deepthi Wickremasinghe, Joanna Schellenberg

**Affiliations:** IDEAS Project, London School of Hygiene & Tropical Medicine (LSHTM), UK

**Keywords:** Decision-making, developing countries, framework, health system

## Abstract

Low-resource settings often have limited use of local data for health system planning and decision-making. To promote local data use for decision-making and priority setting, we propose an adapted framework: a *data-informed platform for health* (DIPH) aimed at guiding coordination, bringing together key data from the public and private sectors on inputs and processes. In working to transform this framework from a concept to a health systems initiative, we undertook a series of implementation research activities including background assessment, testing and scaling up of the intervention. This first paper of four reports the feasibility of the approach in a district health systems context in five districts of India, Nigeria and Ethiopia. We selected five districts using predefined criteria and in collaboration with governments. After scoping visits, an in-depth field visit included interviews with key health stakeholders, focus group discussions with service-delivery staff and record review. For analysis, we used five dimensions of feasibility research based on the TELOS framework: technology and systems, economic, legal and political, operational and scheduling feasibility. We found no standardized process for data-based district level decision-making, and substantial obstacles in all three countries. Compared with study areas in Ethiopia and Nigeria, the health system in Uttar Pradesh is relatively amenable to the DIPH, having relative strengths in infrastructure, technological and technical expertise, and financial resources, as well as a district-level stakeholder forum. However, a key challenge is the absence of an effective legal framework for engagement with India’s extensive private health sector. While priority-setting may depend on factors beyond better use of local data, we conclude that a formative phase of intervention development and pilot-testing is warranted as a next step.

## Introduction

Key Messages

- To promote the use of local data for decision-making and priority setting we propose an adapted framework known as a data-informed platform for health (DIPH).
- We report the feasibility of establishing a DIPH in the context of district health systems in India, Nigeria and Ethiopia, using five dimensions: technology and systems, economic, legal and political, operational and scheduling feasibility.

Low-resource settings often make only limited use of local data for health-system planning and decision-making (Simba and Mwangu 2004). Key challenges include data quality, professional expertise, information-system infrastructure, robustness of technology and a culture of evidence-based decision-making (Nnaji *et al.* 2010; Maokola *et al.* 2011; Qazi and Ali 2011). Data on the contributions of private- and social-sector stakeholders are not readily available, in spite of those stakeholders being major service-providers in some settings (Hanson and Berman 1998; Raban *et al.* 2009). Timely sharing of information might reduce duplication of effort and ensure that resources are not wasted.

Shared data could empower local decision-making, and would reposition health-service delivery in line with available resources and community health needs, although priority-setting depends on other factors too (Hipgrave *et al.* 2014). The Health Management Information System (HMIS) reflects health-facility utilization and performance: local programme staff can report on human and physical resources; non-governmental organizations (NGOs) may have data on community-based activities; and certain private service providers have information on service provision. Yet there is little published evidence of information being brought together at district level (Newell 1989; Wickremasinghe *et al.* 2016). There have been several attempts to bring private- and public-sector data together for better decision-making (Bryce *et al.* 2005, 2011; Asante and Zwi 2007; Victora *et al.* 2011), including the National Evaluation Platform (Johns Hopkins Bloomberg School of Public Health 2016). This framework guides data-coordination for evaluation and policy-making, bringing together data from the public and private sectors that could influence maternal and newborn health. Implemented at the district level, this approach could improve service planning and co-ordination, but would rely on local health departments and other service providers having ownership of assured data-sharing mechanisms. 

**Figure 1.**
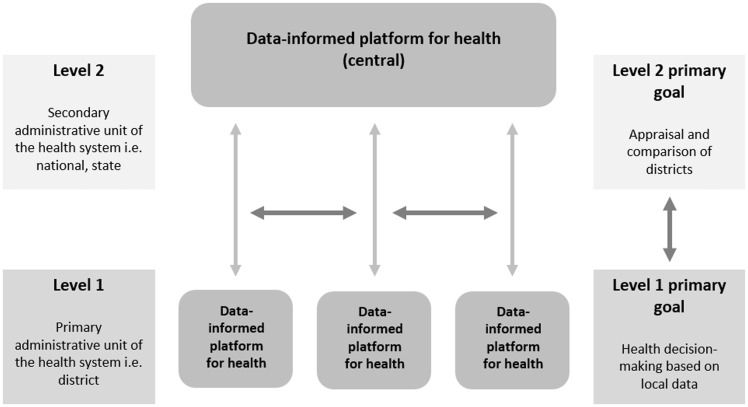
Data-informed platform for health framework

In this series of papers, we propose a data-sharing platform through the district health administration—a *data-informed platform for health* (DIPH)—to guide co-ordination, bringing together key data from the public and private health sectors on inputs and processes, including service delivery, that could influence maternal, newborn and child health (MNCH). District health managers need a robust strategy for resource allocation and delivery of MNCH services. The primary aims of the DIPH are to promote the use of local health programme data for decision-making, priority-setting and planning at the district health administration level; and appraisal of MNCH services and programmes (Figure 1).

The viability of DIPH in a given context is based not only on the availability and sharing of data at the district level but also on several other factors, including the culture of democratic governance, decentralization and public–private partnerships. The DIPH would derive data—a primary prerequisite for objective decision-making—from diverse private- and public-sector health organizations. Governmental and non-governmental service providers would meet in a regular forum to share data in a systematic manner, and to use the resulting information as a tool in priority-setting for resource allocation and needs-assessment for the acquisition of funds.

Here we describe implementation research to assess the feasibility of establishing the DIPH in the context of district health systems in five districts of India, Nigeria and Ethiopia. This was carried out with the broader aim of informing the development, testing and the scaling up of the DIPH intervention. We show results from each setting as well as a comparison across the three geographies. The remaining papers in the series provide evidence on other aspects of the use of district-level data for decision-making. The second paper is a systematic literature review of district decision-making for health in low-income settings, with a particular focus on identifying good practice in formal health-system decision-making at the district level in terms of linking with HMIS data; priority-setting; consensus-building among stakeholders; resource allocation in the context of centralized versus decentralized health systems; and follow-up on the implementation of decisions (Wickremasinghe *et al.* 2016). The review reports that these effective practices happen discretely and in various combinations, and that there is potential to bring them together under a DIPH ‘umbrella’. The third paper presents potential data sources using the World Health Organization’s health system building block framework, and shows the considerable potential of HMIS data at district level in India and Ethiopia (Bhattacharyya *et al.* 2016). The final paper in the series presents prospects for engaging the private sector in sharing health data and making collaborative decisions at the district level in India (Gautham *et al.* 2016).

## Methods

**Figure 2.**
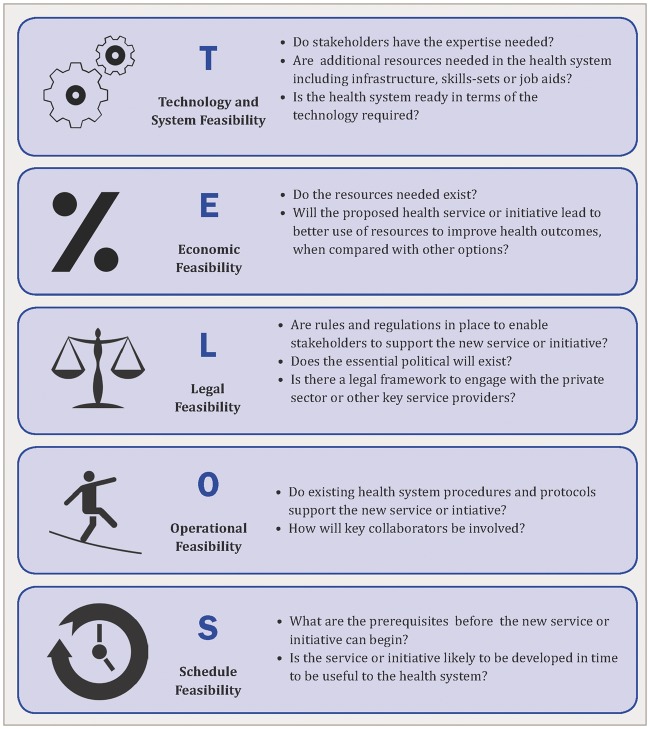
TELOS framework: guide questions for feasibility research

The study is based on the TELOS framework, derived from the Greek philosophy of teleology, the study of the nature or intentions of a plan or object. This concept is used in business and management to assess the feasibility of a new service, programme or initiative (Taylor 2007). To the best of our knowledge it has never been used in health or health-systems research. Feasibility studies in health-systems research focus on opportunities and threats, looking at proof of concepts, and precede technical development and pilot-testing. TELOS uses five dimensions of feasibility research: *T*echnology and *S*ystems, *E*conomic, *L*egal and *P*olitical, *O*perational and *S*cheduling feasibility. Typical guiding questions are illustrated in Figure 2.

The primary use of the framework is as a guide to identify the foundational elements of feasibility for the DIPH and to review information on the availability of these elements. This leads to a discussion of relative feasibility in specific health-system contexts. It is important to highlight that TELOS includes objective elements of decision-making, while in actual decision-making subjective elements also play a major role, including personal or socio-cultural values, weighing up of pros and cons, and the degree of risk-taking to be built in.

Field work for this multi-country study was conducted, in 2012 and 2013, in Uttar Pradesh (UP), India; Ethiopia and North-East Nigeria. The countries and states were chosen because they were focus areas for the MNCH investments of the Bill & Melinda Gates Foundation, due to their high maternal and newborn mortality rates in combination with large populations.

The data collection for feasibility assessment was both cross-sectional and descriptive, and used mixed qualitative methods to triangulate findings, including scoping visits; key informant interviews and participation in planning the inquiry; interviews with service providers; participant observations; and document reviews of health-service records and information systems, district health plans and other relevant reports. Assessment was primarily at district level—the lowest administrative unit of health-system management with the potential to make independent decisions about health service delivery. In Ethiopia, this was the *woreda*, and in Nigeria the Local Government Area (LGA). Throughout this paper we use the term ‘district’. The terms used for administrative levels across the three study settings are given in Table 1. It is important to mention that these terms do not represent equivalence in terms of population size: for example, a block in India may have a larger population than a *woreda* in Ethiopia.

**Table 1.** Terms for administrative levels across three study settings

	UP, India	Ethiopia	North-East Nigeria
1	State	Region	State
2	Division	Zone	
3	District	Woreda	Local Government Area
4	Block	PHCU	Ward
5	Community/village (sub-centre)	Kebele (health post)	

The study was led by the London School of Hygiene and Tropical Medicine, UK. In each country national-level counterparts facilitated both introductions at state level and the work at district level: these were the Public Health Foundation of India, JaRco Consulting, Ethiopia and Health Hub Limited, Nigeria. The steps of the feasibility study along with research methods used are outlined below.

*Official permission and collaboration of the state government*. At the initial meeting with state-government officials the premise and significance of the assessment were explained. Based on their approval and collaboration, a detailed plan was finalized.

*Selection of the districts*. The study districts were selected based on three criteria: (1) variability in local governance, in terms of level of engagement of the district-health administration and performance of health facilities; (2) non-contiguous, so as to minimize their influence on each other and (3) accessible (within a 1-h drive of the state capital), to make the research process manageable. The selected districts were Unnao and Sitapur in Uttar Pradesh, India; and Dendi and Baso in Ethiopia. Because of the security situation, our study was limited to one district, Shongom, in Gombe State, North-East Nigeria.

*Scoping visit*. This was to make contact with the district-health administration and identify potential key informants in the public- and private-health sectors. In close consultation with the District Health Officer and with the solicited co-operation of facility staff, we selected better- and worse-performing facilities at primary-care level in terms of regular availability and quality of services, as well as the district hospital.

*In-depth field visit*. We conducted a two-phase, in-depth field visit using teams of three to four researchers. In the first phase, the team focused on public-health facilities at primary and secondary level: primary facilities provided care in the community for people making a first contact to a healthcare worker for preventive or curative care; secondary facilities provided specialized care upon referral by a primary care provider. In the second phase, the team focused on the private, not-for-profit sector (NGOs), and on private, for-profit service providers, who were registered with the district administration. The field team comprised researchers from the lead institution and from the country-level collaborating institutions with expertise in health-system research, epidemiology, demography and qualitative research. The field visit included: key informant interviews with members of health-system administrations and health-NGO representatives; group discussion with clinical staff of health facilities; and finally, a review of available data and records.

*Key informant interviews*. We carried out semi-structured interviews in order to understand the structure of the health system, co-ordination among stakeholders, data flow and the local context. On average, four to five individuals were identified and interviewed in each district, including the District Health Officer, the person in charge of the district hospital and the district coordinators of key NGOs playing a role in MNCH services.

*Service-delivery staff interviews*. Group discussions were conducted with clinical staff at secondary- (e.g. district hospital) and primary-care-health facilities (e.g. health post, sub-centres, dispensaries), and community-level frontline workers were interviewed in each selected district. The group discussions gave an understanding of the health service, especially in relation to MNCH, and to inter-sectoral coordination among the public-health, private-health and non-health sectors. The details of study participants across the three study settings are given in Table 2.

**Table 2.** Study participants

Inquiry type	Respondent type	India	Ethiopia	Nigeria
In-depth interviews	State level heath ministry representatives	7	6	10
	District health administration representatives	5	4	3
	NGO representative at state and district level	4	4	1
Group discussions	Primary care level clinical staff	4	4	2
	Secondary care level clinical staff	4	4	1
	NGO staff directly involved in service provision	2	2	1

*Record and document review*. To assess the status of service-delivery, record-keeping and data-management operations the review focused on (1) HMIS agreed framework and indicators; (2) the previous year’s district-planning documents; (3) service and administration records maintained at the health-facility level; and (4) NGO reports. Our country-level collaborating partners were instrumental in compiling and assessing these documents.

A list of initial key informants was drawn up in collaboration with the country level collaborating institutions and the state ministry of health. Requests for additional informants were elicited from the original group of interviewees, leading to a snowball sampling process and hence variability in the number of respondents in each setting.

The interview guides, record and document review forms were developed by one of the authors (B.A.) and pretested in Nigeria, and subsequently adapted in India and Ethiopia. The interview guides evolved as new themes emerged during data collection. Primary areas of enquiry were based on the five key components of the TELOS framework.

We captured data using detailed field notes, and analysed information according to a framework approach involving both *a priori* defined components of TELOS and emerging themes. Subsequently, we linked data from key informants and facility records and health-system document reviews (Ward *et al.* 2013; Green and Thorogood 2014). Data analysis was performed by the authors to ensure conceptual clarity and coding consistency across the three contexts. Finally, we summarized relative readiness to implement a DIPH in each country according to the TELOS feasibility framework. The grading was agreed among the study researchers, after critical appraisal of results across the three settings and ranged from sufficient feasibility ( +++) to nil feasibility (−). This reflects variations in each TELOS framework component across three settings as well as across all the components of the TELOS framework within the study sites in UP, India; Ethiopia and North-East Nigeria.

### Ethics

Ethical approval was obtained from the corresponding author’s institute, the Indian Council of Medical Research, the Independent Review Board (IRB)—SPECT-ERB and the Health Ministry Screening Committee in India, the Federal Ministry of Health and the Ministry of Science and Technology in Ethiopia, and Regional Health Bureaus in Amhara, Oromiya and Tigray, in Ethiopia, the Federal Ministry of Health Abuja, Nigeria, and the State Ministry of Health Gombe State, Nigeria.

## Findings

### India

Uttar Pradesh has a population over 200 million and is the most populous state of India. Health status, particularly MNCH, is among the worst in the country (Registrar General of India 2013, 2014; UNICEF 2014). Despite financial incentives, only 46% of deliveries are facility-based (International Institute for Population Sciences 2010). About 90% of health care sought for acute illness, and 80% for chronic illnesses, is from formal and informal private providers, and international and local NGOs either provide health services directly or give support to government. In contrast, only 31% of facility births occur in the private sector in the state of UP (Government of India 2013). Overall, the private sector is marred by a culture in which health-record-keeping and information-sharing are lacking. However, it is important to highlight that preventive and promotive primary MNCH healthcare services are the forte of public health services.

To improve the quality and acceptance of health services, the National Health Mission (NHM, formerly known as the National Rural Health Mission) introduced numerous reforms in 2005, including a cash incentive scheme (Janani Suraksha Yojana—JSY) to encourage women to give birth in health facilities, supported by village-based front-line health workers—Accredited Social Health Activists (ASHAs). Integrated Child Development Services, implemented by the Ministry of Women and Child Development, provide preventive and promotive health care, and produce vaccination and nutritional data. The health system is guided by national standards and staffing norms for primary and secondary care, operational at district level. Administratively, district health services are headed by the Chief Medical Officer (CMO), and additional CMOs are responsible for specific district health programmes, such as nutrition and vaccination. The most peripheral health facility is a sub-centre at village level, staffed by an Auxiliary Nurse Midwife (ANM) and supported by ASHAs. Sub-centres link to Primary Health Centres which provide services for up to 30 000 people. For every four Primary Health Centres there is a Community Health Centre (CHC), providing secondary health care, generally at block level. CHCs link to a district hospital, providing more specialized care.

*Technology feasibility*. The primary data sources for MNCH at community level are ANMs and ASHAs, supplemented by service provision data from Primary Health Centres and CHCs. Through NHM, there is an electronic data entry at CHCs for the Mother-and-Child Tracking System and JSY. However, these data are passed to divisional level (an administrative unit comprising a few contiguous districts) without being processed or used at district level. In parallel, paper-based HMIS reports are prepared and, with limited quality checks, passed on to the Health Directorate (a theme-specific section of the Health Ministry with its own director and implementation team, e.g. Directorate of Nutrition). The data quality of the electronic system is considerably better, but has limited acceptability because it is collected under the federally funded NHM, rather than the state level health directorate.

*Economic feasibility*. NHM aims to improve quality across the health sector, with a focus on access for pregnant women and children. Considerable financial and technical resources are available at district level to improve infrastructure, increase service utilization and generate quality data.

*Legal and political feasibility*. Private for-profit hospitals and medical centres must register with the district health administration, but have no legal requirement to submit data. In the private non-profit sector a mechanism exists for sharing data, but this is primarily above district level.

*Operational feasibility*. The use of health data at district level is primarily limited to micro-planning on vaccination. District macro-level annual planning is usually based on projection of figures from the previous year’s plan. NHM has introduced the District Health Society, with monthly meetings chaired by the District Magistrate and attended by CMOs, staff from Integrated Child Development Services and from NGOs. Its primary purpose is to provide support for planning, including resource distribution and regulation enforcement. There is limited participation by the private for-profit sector and scant use of data in planning.

*Schedule feasibility*. Most critical elements of the DIPH are already in place: credible data, technical expertise, financial and technical resources, and a suitable forum such as the District Health Society. To schedule the DIPH, minor infrastructure changes are needed, as well as a process for decision-making by the District Health Society based on health data.

### Ethiopia

Ethiopia is the second most populous country in sub-Saharan Africa, and has achieved the Millennium Development Goal (MDG) for under-five child mortality. However, maternal mortality is among the world’s worst, with 420 deaths per 100 000 live births (World Health Organization 2014). We conducted this study in the agrarian regions, which occupy 60% of the land and are home to 70% of the population. The private health sector is growing, but accounts for a very small proportion of general health care. NGOs active in health include Save the Children and ChildFund.

The health system under the Ethiopian Health Sector Development Plan has a major focus on primary care. Within each district, there is generally a single district hospital for specialized care, and three to four Primary Health Care Units (PHCUs) which provide integrated, community-based preventive and basic curative services. Each PHCU has three components, linking together referral and supportive supervision functions: the Health Centre, Health Post, the Health Development Army (HDA). The Health Centre is the primary referral unit for every five health posts and provides secondary care. The Health Post serves a population of up to 5000 and is staffed by two female Health Extension Workers (HEWs), who spend up to 80% of their time on outreach services involving hygiene, sanitation, infectious disease prevention, health education and family welfare. HEWs are supported in turn by the HDA—health volunteers, one woman selected from every five households, who provide their neighbours with health education and encouragement to change health behaviours. There is a national shortage of secondary and tertiary hospitals.

*Technology feasibility*. Despite minimal use of technology, data collection is targeted, with HMIS data limited to 108 well-defined indicators, which are primarily MNCH-focused. The District Health Office prepares a monthly report to the zone (an administrative unit comprising a few contiguous districts) based on key indicators; and a detailed report is prepared on a quarterly basis. Information flows slowly and there is limited expertise in synthesizing information at district level. An acute shortage of technical and administrative staff means that staff are overburdened, and technical staff are often required to carry out tasks originally planned for those with more training (task shifting).

*Economic feasibility*. Ethiopia is among the poorest countries in the world, with a United Nations Gross Domestic Product ranking of 86. There is a general lack of health resources. At district level, there are two main relevant forums: the elected Council approves the allocation of the district-level budget, and the Cabinet—with representations from all major departments, including health, education and agriculture—is responsible for budget preparation. The struggle for equitable resources for health, including for MNCH, at district level has both political and financial causes, the latter exacerbated by limited use of data to justify an increase in spending.

*Legal and political feasibility*. NGOs and private health providers are tightly regulated and are required to submit monthly reports including service delivery information to the District Health Office. However, they have a very limited role in MNCH service provision because of the general population’s inability to pay.

*Operational feasibility*. District-based planning aims to meet local health needs within the context of national targets. At federal level, a technical working group provides direction and selects indicators based on the Health Sector Development Plan and the MDGs. District-level health management is expected to use the Marginal Budgeting for Bottlenecks Tool and dashboards, but a lack of technical capability leads to limited use.

*Schedule feasibility*. Government administration and financial support are essential for sustainability. A lack of resources, including for the use of technology, creates key challenges. Empowerment of the District Health Office and the Cabinet are prerequisites.

### Nigeria

In 2014, Nigeria was declared Africa’s largest economy (International Monetary Fund 2014). Yet population health lags behind that of poorer African nations. The maternal mortality ratio is 576, and under-five mortality is 128 deaths/1000 live births (National Population Commission Nigeria and ICF International 2014). The North-East region has limited development in social and health sectors, and security is fragile. Health indicators are generally worse than in the rest of the country. 

The Nigerian health system has two distinct features: (1) the federal government is directly responsible for delivering tertiary care in each state, while secondary and primary care are managed by state health ministries, which leads to problems with continuity of care; and (2) both state and district government receive formula-based funds for health from the federal government. These are not earmarked for specific issues and there is no accountability for their use.

At district level, the health system includes the Health Post (< 500 people) and the Primary Health Clinic (>500 to ca.10 000 people). These should be staffed by community HEWs (CHEWs), supported by village health workers (as with the HDA in Ethiopia and ASHAs in India). For every 10 000—20 000 population there should be a referral facility—a Primary Health Centre, staffed by nurses and midwives. However, there is a chronic shortage of qualified staff.

*Technology feasibility*. Due to the lack of infrastructure and security, HMIS, including MNCH-specific, data collection and processing capabilities are limited. The flow of information from facility to district and state level is segmented. Essential job aids—such as registers—are scarce. Data are usually compiled manually.

At district level the Department of Primary Health Care collates MNCH data, including antenatal care, nutrition, sanitation, immunization etc., and these are shared at state level, without any integration of primary care data at district level. There is parallel reporting of many streams of data without any coordination or integrated use at district level.

*Economic feasibility*. The health system is on the verge of collapse in some places because of the security situation. In a context of limited resources and minimal accountability, efforts to secure essential supplies and ensure health staff availability take precedence over strengthening systems for data collection and data use at district level.

*Legal and political feasibility*. We found a strong political will to improve the health system and request technical help, but there is a constant challenge to retain high-quality administrative and service provision staff. Local and national NGOs—such as the Society for Family Health—and the National Union of Road Transport Workers (with a scheme responsible for transporting emergency maternity patients) are present, but their operations are not well integrated into the health system and they are limited by legal regulations. The private sector has little prominence due to a lack of recognition in the health system and limited affordability.

*Operational feasibility*. Despite having recommended MNCH indicators under the National Monitoring and Evaluation Framework, and regulatory expectations from the districts and state to provide information, there is no full implementation of procedures.

*Schedule feasibility*. The most vital challenges for the health system at state level in Gombe are the fragile political setting, poor security, and a lack of necessary infrastructure and accountability. The effect is a focus on *ad hoc* service provision measures, rather than building a system to use health data to plan and establish long-term solutions.

A summary of relative findings across all three settings by the TELOS framework is shown in Table 3. This highlights the strengths of the UP-India context in terms of technological and economic feasibility, and its comparability with Ethiopia in terms of the legal and operational feasibility for the DIPH. In contrast, operational realities in Nigeria are such that the feasibility of DIPH would necessitate building basic capacity in health administration and HMIS.

**Table 3.** Summary of feasibility study findings based on TELOS framework from the three geographical contexts

Components	Specific inquiries considered	India	Ethiopia	Nigeria
Technology and systems feasibility	Do stakeholders have the expertise needed for DIPH?How ready is the health system in terms of technology?	+++	+	−
Economic feasibility	Do the resources needed for the DIPH exist?	+++	±	±
Legal and political feasibility	Are the rules and regulations necessary for stakeholders to support the DIPH in place?Does the political will exist to support the DIPH?	++	++	±
Operational feasibility	Do existing health system procedures and protocols support the DIPH?	++	+	+
Schedule feasibility	Are the prerequisites needed in place prior to executing the DIPH?	++	++	±

+++, sufficient;  ++, basic minimum; +, limited; ±, negligible; −, nil.

### Level of decentralized decision-making

All the study areas claim to have decentralized health systems ensuring that local decision-making meets local needs at district level. In reality, practices of autonomous decision-making and health-planning protocols vary. In India, the planning process is primarily from the block and district to the state level, streamlined with the federal-government-funded NHM programme, while in Ethiopia district-level health-planning is guided by national government targets. In Nigeria, state- and national-level guidance to support district-level planning and accountability are not well connected.

### Level of government engagement in the DIPH concept

Across the three geographies, district health officials unanimously reported that the main utility of the platform would be to provide evidence for decision-making and planning. Some district officials said they would not be comfortable using the DIPH as a tool for evaluation because they are too close to service delivery. However, at the higher level—state and sub-state—the DIPH was felt to be an acceptable strategy for both monitoring and evaluation.

Further details of the nature, type and extent of MNCH data based on the content analysis of the HIMS at district level is given in The third paper in this series (Bhattacharyya *et al.* 2016), while the fourth paper presents prospects for engaging the private sector in health-data sharing and collaborative decision-making at the district level in India (Gautham *et al.* 2016).

## Discussion

This study of the district health system across three geographical contexts shows both potential for establishing the DIPH and major challenges. Compared with study districts in Ethiopia and North-East Nigeria, the health system in study districts of UP-India is relatively amenable to the DIPH due to relative strengths in infrastructure, technological and technical expertise, and financial resources, as well as the availability of a district-level forum for stakeholders. However, a key challenge for India—in contrast with Ethiopia—is the absence of an effective legal framework within which to engage with the extensive private health sector, including the informal private sector. On the other hand, a feature of the fragility of North-East Nigeria is a severely damaged health infrastructure, whose improvement needs to take precedence over the introduction of any new initiative such as the DIPH. Consistent across all three geographical contexts is a lack of any standardized processes for data-based decision-making at district level.

The DIPH has potential, as a bottom-up decision-making strategy in the health system, to include important perspectives and information available at district level. The present study has highlighted the relative feasibility of this approach, revealing major obstacles as well as opportunities across diverse contexts. Operationally, the DIPH provides an approach to various types of decision-making in health services, as well as opportunities for contemporary management and analytical techniques such as *dashboards*, to monitor the progress of key performance indicators. (Felkey and Fox 2014) The *balanced scorecard* is a widely recognized strategic planning and management system for improving communications and monitoring the performance of health services against defined goals (Kocakulah and Austill 2007; Behrouzi *et al.* 2014). *Implementation strength* (Miller *et al.* 2014) can be used to decide which resources and activities are needed for health systems to achieve specific coverage targets at district level.

Currently, a number of parallel global efforts are in progress in the field of HMIS and health-data management. For example the Health Information System Programme, India specializes in designing and implementing solutions in health informatics for the public health sector in the Indian states, Bangladesh and Sri Lanka (HISP India 2015). Similarly, Health Information System Programme, Nigeria focuses on improvement in HMIS in the African region (HISP Nigeria 2015). Despite limitations and challenges, the DIPH could complement these efforts by focusing on the better use of data at local level through a platform for collaborative decision-making and action-planning.

The challenges of incorporating the DIPH into district-level health systems can be organized in an adapted three challenge point framework (Guadagnoli *et al.* 2012).

### 1. Complexities of the DIPH process

The complexity of the DIPH is intrinsic to its strength, i.e. engagement with multiple stakeholders and the use of multiple data sources in a structured decision-making process. In general, the health system in low-income countries is marked by a rigidly structured and hierarchical governance, with limited decision-making authority at district level (Bossert 1998; Nutley *et al.* 2013). A process of collaboration and evidence-based decision-making is a key foundation of effective DIPH implementation: following on from the current study, significant formative work and action research are needed. Although the district is the health system’s lowest major unit of administration and governance in all of the three countries studied, key decisions are made either at a higher level or at district level on an *ad hoc* basis. The second paper in this series is a systematic literature review on standardized decision-making methods used by district-level health administrators and managers in low-income countries (Wickremasinghe *et al.* 2016).

Another complexity in operationalizing the DIPH lies in engaging with stakeholders—especially NGOs and private-sector agencies—with a key role in health-service delivery. It is important to highlight that, unlike in Ethiopia, the registration of private-service providers with the government in India and in Nigeria does not equate with their participation in district-level HMIS. To streamline collaborative district-health initiatives, NGO and private sector resource contribution should be counted at district level and they should be actively engaged in the decision-making process. We found limited evidence both of data-sharing and of legislation to support public–private collaboration at district level. The fourth paper in the series provides an assessment of the data-sharing potential of the private sector and recommends some strategic action points for engaging with the private sector (Gautham *et al.* 2016).

### 2. Resources and skills needed

The current trend in health data use is either to focus on specific health system functions, or to make limited use of data for vertical programmes. The DIPH would shift focus onto using data for a basic package of preventive and curative services. Sound quality data on health service delivery and population health status would be a key resource, yet the availability and quality of HMIS data are considered incomplete in many low-income countries. The potential of the existing HMIS needs careful evaluation, and the third paper examines district-level data, from both the public and the private sectors, from India and Ethiopia (Bhattacharyya *et al.* 2016).

### 3. Context of implementation

In a low-income context, periodic threats of epidemics, destabilized political systems or major conflicts lead to a deterioration of the health system (Tashobya *et al.* 2014).

To the best of our knowledge this is the first use of the TELOS framework in health, particularly for the feasibility of a health system intervention (Taylor 2007). Overall, the framework has provided a detailed assessment of health system stakeholders, operations and resources, giving a common reference across contrasting contexts. Implementation research has a crucial role in innovation to improve health systems. Despite established methods for formative and summative research, there is little dialogue on feasibility research, i.e. the generation of evidence on the viability of innovations in health systems. Feasibility studies have been more common in clinical studies and in high-income health systems than in health-systems research in low-income settings. The structured framework we used in this feasibility study for the DIPH may be of general interest in research on systems innovations.

Our study was limited to a small number of purposefully selected districts across three geographical settings; indeed, in Nigeria we were able to assess only one district. However, every effort was made to include representative districts and health facilities in close consultation with the state and district level stakeholders. The level of local engagement in the research could introduce bias in the short term in the sense of limiting access to extremely poor-performing health facilities; however, securing local engagement in and ownership of the research in the long-term is of great value. Feasibility assessment through the TELOS framework primarily focused on identifying tangible elements needed to plan the DIPH, and is a small first step in development of the approach. The next step is a formative phase of intervention development and pilot-testing, which is ongoing in India. The operationalization of any new initiative in a health system is subject to socio-economic and political developments, and needs ongoing repositioning if it is to be successful.

## Conclusion

The study adopted the TELOS framework approach to assess the feasibility of the DIPH across three geographical settings in UP, Ethiopia and North-East Nigeria. In conclusion, first, local stakeholders viewed the DIPH as a potentially valuable strategy for enhancing the use of local data in collaborative and effective decision-making. Secondly, and based on the five feasibility criteria of technology and systems, economic, legal and political, operational and scheduling, India offers the strongest eligibility for implementing the DIPH, followed by Ethiopia, but there are also major challenges in all the settings we studied. Thirdly, the lack of standardized, participatory decision-making among stakeholders was common across all contexts. Support measures—including technical and management capacity building—are needed to varying degrees across the health systems.
